# Experimental investigation of a nano coating efficiency for dust mitigation on photovoltaic panels in harsh climatic conditions

**DOI:** 10.1038/s41598-024-72772-7

**Published:** 2024-10-03

**Authors:** Asmaa Ahmed, Mohamed Elsakka, Yasser Elhenawy, Ahmed Amer, Amr Mansi, Mohamed Bassyouni, Mamdouh Gadalla, Ahmed Refaat

**Affiliations:** 1 Mechanical Power Engineering Department, Port Said University, Port Said, Egypt; 2 Energy Research and Studies Centre, Port Said University, Port Said, Egypt; 3 Chemical Engineering Department, Port Said University, Port Said, Egypt; 4 Centre of Excellence in Membrane-Based Water Desalination Technology for Testing and Characterization (CEMTC), Port Said University, Port Said, Egypt; 5 Electrical Engineering Department, Port Said University, Port Said, Egypt

**Keywords:** PV performance, PV Soiling, Dust mitigation, Nano coating, Solar cells, Solar cells

## Abstract

Dust accumulation on photovoltaic (PV) panels in arid regions diminishes solar energy absorption and panel efficiency. In this study, the effectiveness of a self-cleaning nano-coating thin film is evaluated in reducing dust accumulation and improving PV Panel efficiency. Surface morphology and elemental analysis of the nano-coating and dust are conducted. Continuous measurements of solar irradiances and ambient temperature have been recorded. SEM analysis of dust revealed irregularly shaped micron-sized particles with potential adhesive properties, causing shading effects on the PV panel surface. Conversely, the coating particles exhibited a uniform, spherical shape, suggesting effective prevention of dust adhesion. Solar irradiance ranged from 120 W/m² to a peak of 720 W/m² at noon. Application of the self-cleaning nano-coating thin film consistently increased short circuit current (*I*_*sc*_), with the coated panel averaging 2.8 A, which is 64.7% higher than the uncoated panel’s 1.7 A. The power output of the coated panel ranged from 7 W to 38 W, with an average of approximately 24.75 W, whereas the uncoated panel exhibited a power output between 3 W and 23 W, averaging around 14 W. These findings highlight the substantial potential of nano-coating for effective dust mitigation, particularly in dusty environments, thus enhancing PV system reliability.

## Introduction

Photovoltaic (PV) systems are a promising technology for renewable energy, permitting the conversion of sunlight into electricity. Nevertheless, the widespread implementation of PV systems faces a significant obstacle due to the accumulation of dirt, dust, and additional particulate materials on solar panel surfaces^1,2^. Dust refers to any particles of matter with a diameter of less than 500 µm present in the atmosphere^3^. This can consist of organic and inorganic particles such as soil, pollen, and factory smoke. Therefore, soiling decreases the sunlight reaching PV cells, reducing the system’s power output^4^. In areas with high levels of air pollution, arid temperatures, and agricultural activities, this phenomenon is more severe. Not only does soiling significantly impact the economic feasibility of PV systems but it also affects their contribution to global efforts to transition towards a sustainable energy future^5^. The impact of photovoltaic soiling on the performance and reliability of PV systems has induced extensive research aimed at developing applicable strategies to mitigate these effects^6^. A variety of cleaning techniques, such as robotic cleaning systems and water-based methods, have been developed to minimize soiling losses^7,8^. However, several factors determine the effectiveness of these techniques, including the type and nature of soiling, cleaning frequency, and cost-effectiveness^9^. Moreover, natural cleaning techniques, such as rainfall and dew, can also serve as a restorative approach to mitigating soiling losses. However, these techniques may cause more damage to the panels due to increased corrosion and mineral deposit buildup^10,11^. Another approach involves the development of advanced coatings and surface treatments that prevent or reduce the growth of particulate matter on the surface of PV panels^12^. These coatings can be employed during the manufacturing process or painted onto existing panels. The effectiveness of coatings applied to PV panels depends on a complex interplay of factors. These factors include the type and size of particulate matter present in the environment, and prevailing weather conditions. Broadly, these coatings can be categorized into two main classes: hydrophobic and hydrophilic. Hydrophobic surfaces are characterized by a water contact angle (WCA) greater than or equal to 90° and low surface energy^13^. This design principle allows them to repel water and moisture effectively. By reducing the surface energy of the PV panel, these coatings cause water droplets to bead up and roll off the surface, minimizing water stagnation^14,15^. This rolling action helps prevent the accumulation of dust and dirt on the solar cells, thereby mitigating efficiency losses. Common materials used for hydrophobic coatings include silicones, fluorochemicals, and polymers, each offering distinct advantages and limitations in terms of performance and longevity^16,17^. In contrast, hydrophilic surfaces exhibit a WCA of less than 90° and possess high surface energy^18^. These coatings attract and spread water droplets into thin films across the panel surface^19,20^. This thin film can act as a self-cleaning mechanism, sweeping away dust and dirt particles as the water sheet evaporates. Titanium dioxide is frequently employed for hydrophilic coatings due to its high absorbency and photocatalytic properties^12^. Under UV light exposure, it can even break down certain organic materials deposited on the panel surface. Beyond the basic hydrophobic and hydrophilic categories, there exist extreme surface wettability classifications. Superhydrophobic surfaces exhibit exceptionally high-water repellence, with WCAs exceeding 150°.These surfaces often display self-cleaning properties due to the formation of tiny air pockets trapped between water droplets and the surface texture. Conversely, superhydrophilic surfaces demonstrate a remarkably strong affinity for water, characterized by WCAs below 5°^21,22^. Materials like certain nanostructured surfaces fall within this category. Researchers are actively exploring a wider range of materials to create both superhydrophobic and superhydrophilic surfaces for PV panel applications. Organic materials are showing promise alongside traditional inorganic materials like zinc oxide and titanium dioxide^23–25^. Both hydrophobic and hydrophilic coatings offer unique advantages in maintaining the cleanliness and efficiency of PV panels, with their specific applications depending on environmental conditions and desired maintenance characteristics. The effectiveness of PV panels hinges on maximizing light absorption on their surfaces. Egypt is one of the locations that receive 9 to 11 hours of sunlight daily on average^26^. Its average global horizontal solar irradiation is 2260 kWh/m^2^/year, making it an excellent location for electricity production^27^. As per the Egyptian New and Renewable Energy Authority^28^, Egypt has set a target to increase the installed photovoltaic capacity to account for 22% of the entire electricity generation by 2035. However, due to the nature of the Egyptian dusty climate, low rainfall, and high wind speeds, PV panels are susceptible to dust accumulation^29^ which adversely affects the power produced. According to a previous study^30^, PV soiling is expected to decrease the power output by more than 50% over three months compared to cleaned PV panels. The impact of dust buildup on glass specimens in a particular location in Cairo was examined by Elminir et al.^31^. However, the authors did not assess the influence of soiling on PV performance, which is a critical factor for understanding PV yield. An indoor study was conducted by Menoufi et al.^30^ to compare the performances of two PV panels: dusty and clean in Beni-Suef, Egypt. The results revealed a significant decrease in the performance of the dusty panel compared to the cleaned one. Nevertheless, the authors did not suggest a dust mitigation technique to improve the PV performance. Al-badra et al.^32^ examined the impact of using a hydrophilic nano-coating as a soiling reduction technique. The main component of this material is silica which is known for its hydrophilic properties. The outdoor experimental measurements were conducted for six weeks. The authors mentioned that the periodic cleaning of the PV panels has decreased by 50% per month as a result of using this soiling reduction method. The effect of silicon dioxide nano-coating on PV electrical efficiency was examined by Alamri et al.^33^. The results demonstrated that the power output improved by 15% relative to that of the dusty panel. The literature reveals a research gap in assessing the performance of nanocoatings as soiling reduction techniques for PV panels installed in Egyptian climatic conditions, as dust properties are site-specific and mainly depend on geographical location, environmental factors, and meteorological conditions. Considering the economic and environmental implications of photovoltaic soiling, further research is urgently required to develop effective strategies to mitigate its effects on PV systems’ performance and reliability. A recent study demonstrated that the deposition of silicon-rich oxide onto silicon PV panels could increase energy conversion efficiency. The oxide film absorbs radiation in the near UV range (i.e. ~ 300 nm) and re-emits radiation in the red-light region which falls within the absorbable wavelengths of silicon PV panels^34^. Moreover, when the metal oxide film is subjected to UV illumination, the surface energy is boosted. The increase in surface energy shifts the water-solid interface from the Cassie state (i.e. low interfacial area) to the Wenzel state (i.e. promoted interfacial area)^35^. Nevertheless, it should be emphasized that self-cleaning hydrophilic coatings compete substantially with hydrophobic coatings in lab and rural environments. Hydrophilic coatings can sustain their performance up to 25 years of operation compared to the shorter life expectancy of hydrophobic coatings which lose their functionality in three to four years^36^. This research aims to investigate the effectiveness of a synthesised self-cleaning nano-coating thin film^37^ as a soiling reduction technique for PV panels installed in Port Fouad, Port Said, Egypt [31^o^ 16’ N, 32^o^ 18’ E]. The material is applied for cleaning vehicle windscreen. However, its application on PV panels has not been reported in the literature. Two PV panels were installed and compared; one coated and one uncoated. The effect of using this solution on PV performance parameters and durability was investigated and reported in this research, contributing to the worldwide shift towards a sustainable energy future.

## Materials and methods

### Experimental setup

**Fig. 1:**
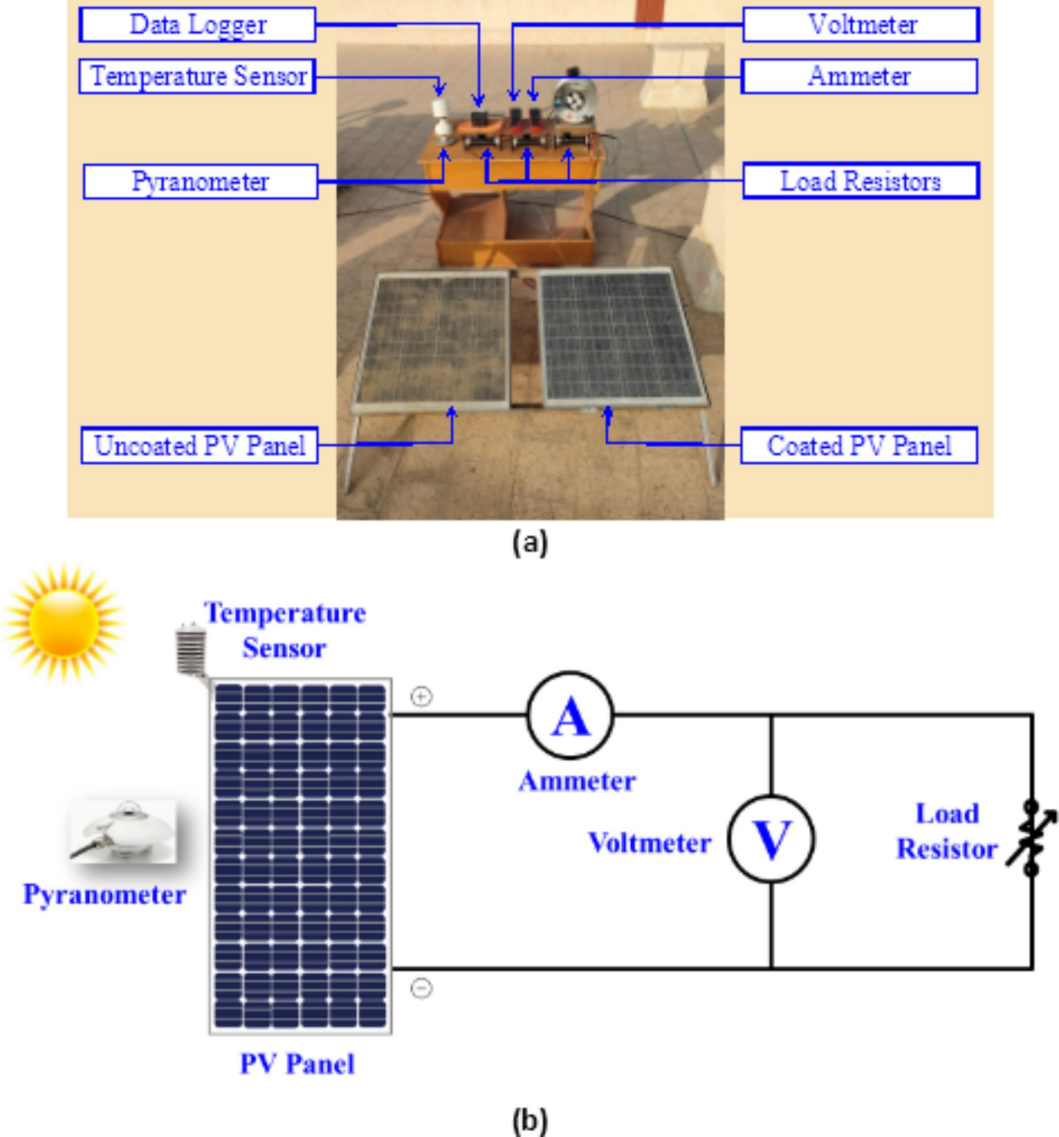
Experimental setup (**a**) Photograph of the system and (**b**) Schematic of the measured circuit.

Several components are interconnected to investigate the performance of coated and uncoated (dusted) PV panels as shown in Fig. 1. The PV panel, which is tilted at 30^o^, representing the core element of the system under study, is integrated within the circuit configuration. The characteristics of the PV Panel are shown in Table 1. The PV Panel is connected in parallel with a load resistor, thereby facilitating the precise quantification of current and voltage characteristics as introduced in Fig. 1b. By employing this parallel connection, the electrical behaviour of the PV panel can be systematically examined, allowing for a comprehensive understanding of its performance attributes. To facilitate accurate and reliable measurements, the experimental setup incorporates two indispensable instruments: a UNI-T voltmeter and a UNI-T ammeter. These instruments are positioned within the circuit to enable the precise monitoring and quantification of voltage and current values, respectively. The voltmeter, known for its high precision and sensitivity, is employed to gauge the potential difference across the terminals of the PV panel. Simultaneously, the ammeter, characterized by its ability to ascertain current flow, is utilized to determine the magnitude of electric current flowing through the circuit. By incorporating these instruments, the experimental setup ensures accurate data acquisition, thereby facilitating robust analysis and interpretation of the PV panel’s electrical characteristics.

**Table 1:** Characteristics of the PV panels.

Cell type	Polycrystalline
Cell size	156 × 63 mm
Panel dimensions	665 × 668 × 35 mm
Maximum power ($\:{\varvec{P}}_{\varvec{m}\varvec{a}\varvec{x},\varvec{s}\varvec{t}\varvec{c}}$)	60 W
Maximum power voltage ($\:{\varvec{V}}_{\varvec{m}\varvec{p}}$)	19.01 V
Maximum power current ($\:{\varvec{I}}_{\varvec{m}\varvec{p}}$)	3.16 A
Open circuit voltage ($\:{\varvec{V}}_{\varvec{o}\varvec{c}}$)	22.61 V
Short circuit current ($\:{\varvec{I}}_{\varvec{s}\varvec{c}}$)	3.38 A
Module efficiency	13.71%
Temperature coefficient of ($\:{\varvec{V}}_{\varvec{o}\varvec{c}}$)	-0.38%/ °C
Temperature coefficient of ($\:{\varvec{I}}_{\varvec{s}\varvec{c}}$)	0.04%/ °C
Temperature coefficient of ($\:{\varvec{P}}_{\varvec{m}\varvec{a}\varvec{x}}$)	-0.47%/ °C

In recognition of the importance of temperature in determining the performance of PV systems, a radiation shield is carefully employed to mitigate the undesirable effects of direct solar radiation on the ambient temperature measurements. The radiation shield, acting as a protective barrier, effectively isolates the temperature sensor from direct solar radiation, thus minimizing potential measurement errors induced by solar heating. By ensuring a controlled and shielded environment, the experimental setup ensures accurate temperature readings, which are critical for comprehending the elaborate interplay between temperature variations and the electrical behaviour of the PV panel.

Moreover, to investigate the impact of incident solar radiation on the performance of the PV panel, a WeatherMeasure Weathertronics pyranometer is integrated into the experimental setup. This instrument serves as a reliable means of measuring the incident solar radiation on the PV panel. Consequently, the integration of the pyranometer within the experimental setup allows for a comprehensive assessment of the PV panel’s response to varying levels of solar irradiance. The combination of these components within the experimental setup presents a robust platform for the systematic evaluation and analysis of the PV panel’s electrical characteristics and the influence of incident solar radiation on its performance.

### Synthesis of the nano-coating

**Fig. 2:**
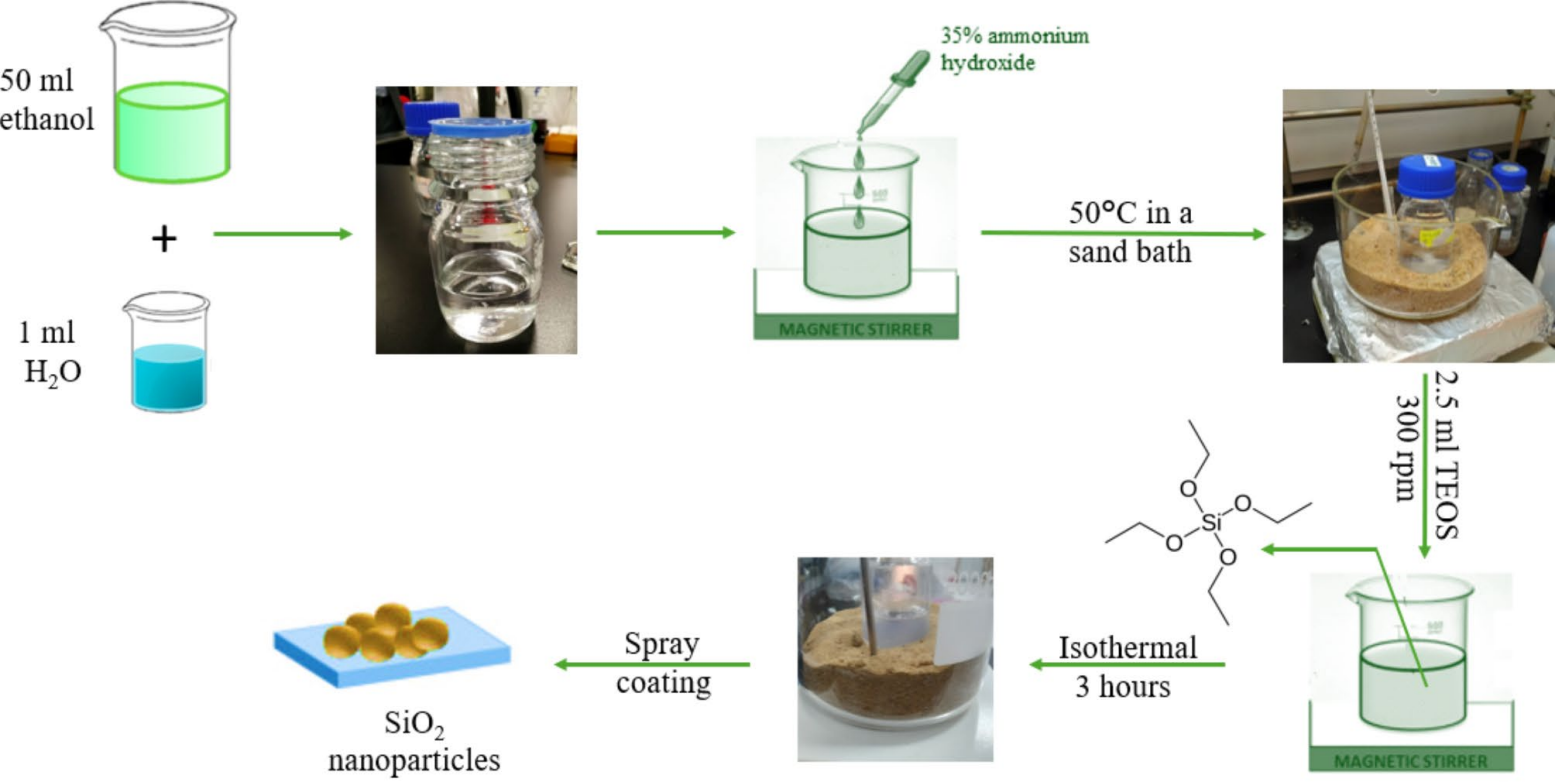
Synthesis procedure of the nano-coating utilized in this study.

The synthesis procedure for the nano-coating is summarized in Fig. 2. Initially, 50 ml of ethanol, acting as the solvent, was added to a glass bottle, followed by the addition of 1 ml of deionized water. Subsequently, 2.2 ml of 35% ammonium hydroxide was added to control the pH of the solution and catalyse the reaction. The mixture was then heated to 50 °C in a sand bath for enhanced temperature control. Finally, 2.5 ml of tetraethyl orthosilicate was added to the mixture, which was stirred at 300 rpm for 3 h.

### Application of the nano-coating

Prior to the application of the self-cleaning nano-coating thin film onto the solar panel, it was crucial to ensure the panel’s cleanliness. Therefore, a rigorous procedure was followed. Firstly, an inspection of the PV panel was conducted to identify any damages or defects. Subsequently, lint-free cloth was utilized to carefully clean the panel and remove any accumulated dust particles. Following the cleaning process, the PV panel was positioned within a controlled environment featuring optimal temperature and humidity conditions, deemed suitable for the subsequent application of the coating. Employing a spray gun, the self-cleaning nano-coating thin film was uniformly and evenly applied onto the entire surface of the PV panel, with utmost attention given to avoiding excessive coating thickness or uneven distribution. The coating was applied batch-wise, and the optimum spraying batch was 5 sprays/ft^2^. Then, the coated panel underwent a natural drying phase, ensuring the formation of a durable coated layer. Once the appropriate drying period had elapsed, the coated PV panel was ready for testing under outdoor conditions. The PV panels were exposed to outdoor conditions for 10 months. The electrical performance of both panels was evaluated, compared, and analysed under identical environmental conditions.

### Characterization of the nano-coating and dust

Extensive characterization was carried out to investigate the characteristics of the dust particles and the coating. The morphology of the deposited nano-coating and the dust was imaged by field-emission scanning electron microscopy (FE-SEM model TESCAN MIRA, Czech Republic) with a landing energy of 5 keV. Dust composition is an important factor in determining its adhesion characteristics. Therefore, elemental analysis of the dust was carried out using energy-dispersive X-ray spectroscopy (EDX model Oxford Xplore, United Kingdom) with 0.01 wt% sensitivity to establish the elemental composition of the dust particles. The nano-coating formula was not made available by the manufacturer. Therefore, Fourier-transform infrared spectroscopy (FTIR model BRUKER ALPHA II, United States) along with the EDX analysis were used to determine its constituents. The average particle size of the dust and the nano-coating was measured by dynamic light scattering using a zetasizer instrument (Malvern Panalytical zetasizer pro, United Kingdom). Moreover, the zeta potentials of the dust and coating were measured in order to investigate the anti-soiling mechanism of the nano-coating. To further investigate the anti-soiling mechanism, the wettability of the deposited nano-coating was determined by measuring the water contact angle on the coated glass surface with an optical tensiometer (KRUSS DSA25S, Germany).

## Results and discussion

### Nano-coating and accumulated dust characteristics

#### Morphology and chemical composition

**Fig. 3:**
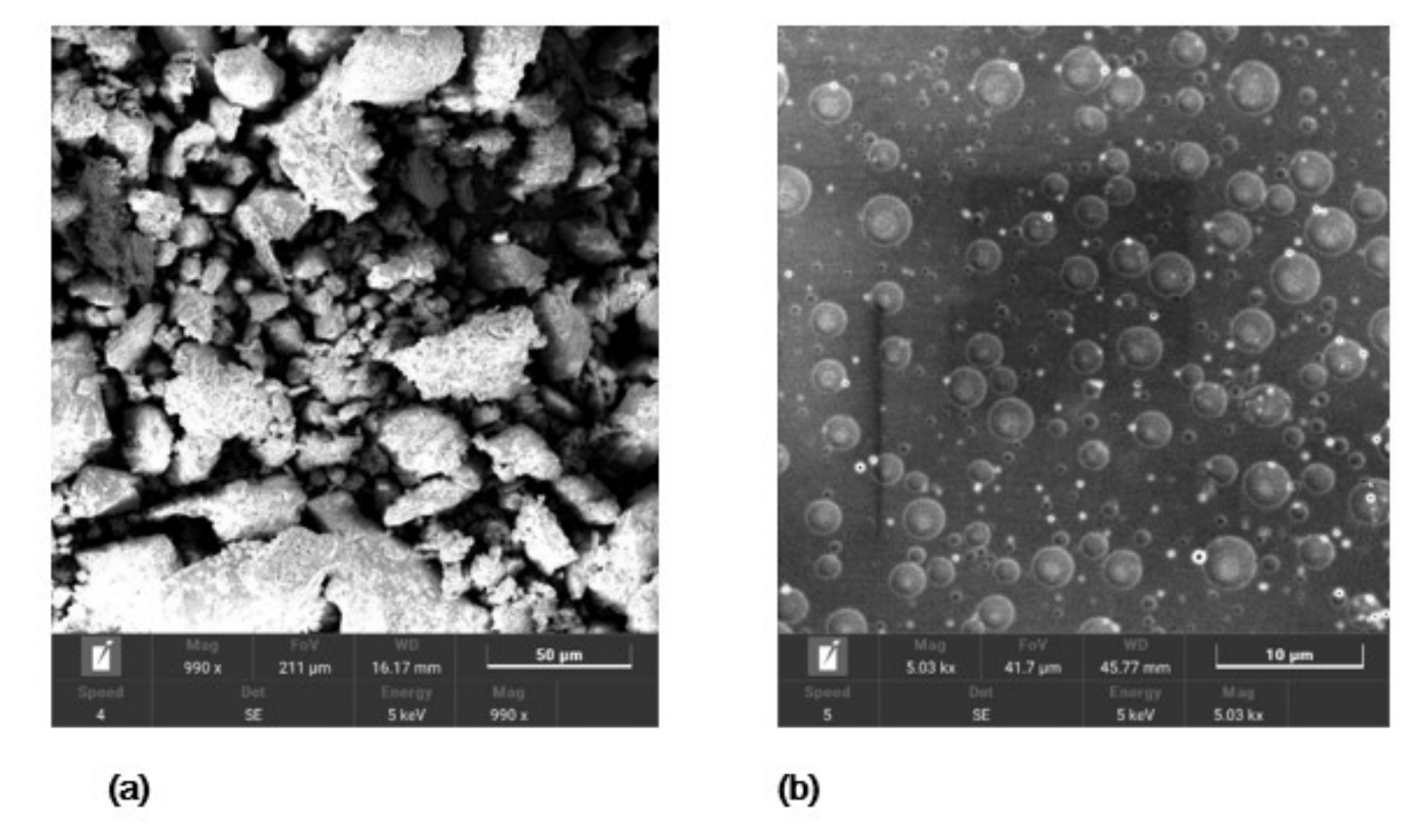
SEM images of the (**a**) dust particles, and (**b**) sprayed coating.

As presented in Fig. 3, The SEM micrographs provided valuable insights into the morphological features of the dust particles and coating. The SEM image of the dust sample revealed the presence of micron-sized amorphous particles. This suggests that the dust particles present in the samples possess a non-uniform and irregular shape, which may contribute to their adhesive properties and potential shading effects on the PV panel surface. In contrast, the SEM image of the coating sample showcased a different morphology. The coating appeared in the form of spherical particles that were homogeneously distributed across the sample surface. The observed spherical shape of the coating particles suggests a uniform and consistent coverage, which may contribute to the coating’s properties and its ability to prevent dust adhesion on the PV panel surface.

**Fig. 4:**
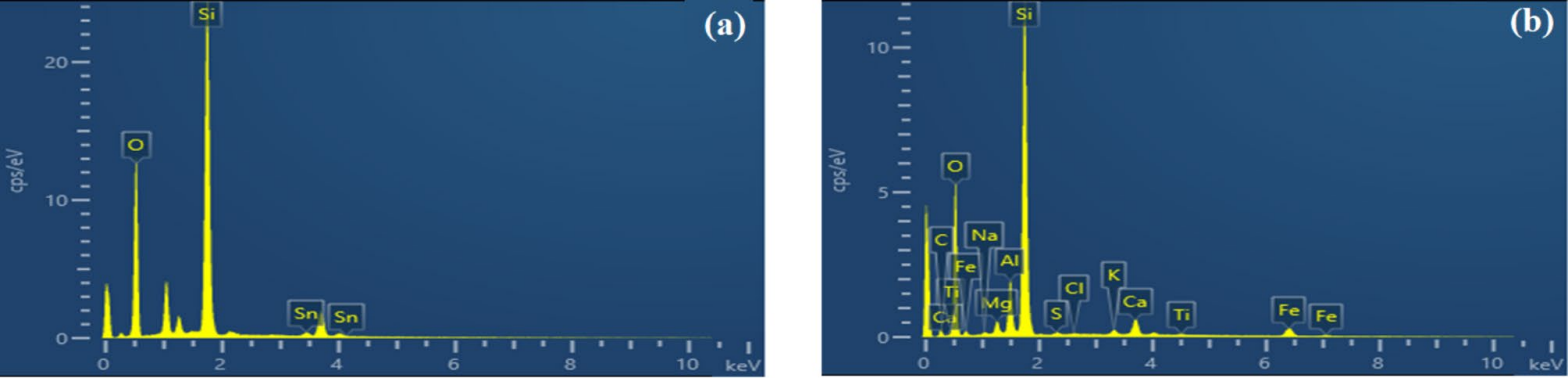
EDX Results of (**a**) sprayed coating composition and (b) dust sample composition.

Figure 4 shows the intensity chart of EDX analysis for the coating and the dust. Oxygen was found to be the most abundant element in the dust sample, constituting 44.73% of the sample’s weight. This high oxygen content suggests the prevalence of oxides and possibly silicates in the dust composition. Silicon, aluminium, and iron are commonly found in mineral dust particles, further indicating the mineralogical nature of the dust sample or the possible contribution of industrial activities. Additional elements detected in relatively lower concentrations include sodium (Na), magnesium (Mg), chlorine (Cl), potassium (K), and calcium (Ca). Their presence may be attributed to the influence of environmental factors, such as air pollution or local soil composition. On the other hand, the composition of the coating sample reveals that oxygen is the major element, representing 50.53% of the weight of the coating. This high oxygen content suggests the presence of oxides in the coating composition, which is consistent with the expected chemical properties of the coating. Also, silicon represents 27.87% due to its desirable properties, such as its ability to resist dust adhesion. The presence of tin is attributed to the glass substrate. A summary of the dust sample and nano-coating compositions is presented in Table 2.

**Table 2:** Summary of the elemental composition of the coating and the dust particles.

Element	Nano-coated glass		Dust particles	
	Wt.%	Atomic %	Wt.%	Atomic %
O	58.4	73.16	44.73	53.24
Si	36.38	25.96	27.29	18.49
Sn	5.22	0.88	–	–
C	–	–	12.91	20.46
Fe	–	–	5.34	1.82
Al	–	–	4.15	2.92
Ca	–	–	2.97	1.41
Mg	–	–	1.05	0.83
K	–	–	0.72	0.35
Ti	–	–	0.27	0.11
Na	–	–	0.23	0.19
S	–	–	0.21	0.12
Cl	–	–	0.11	0.06
Total	100	100	100	100

**Fig. 5:**
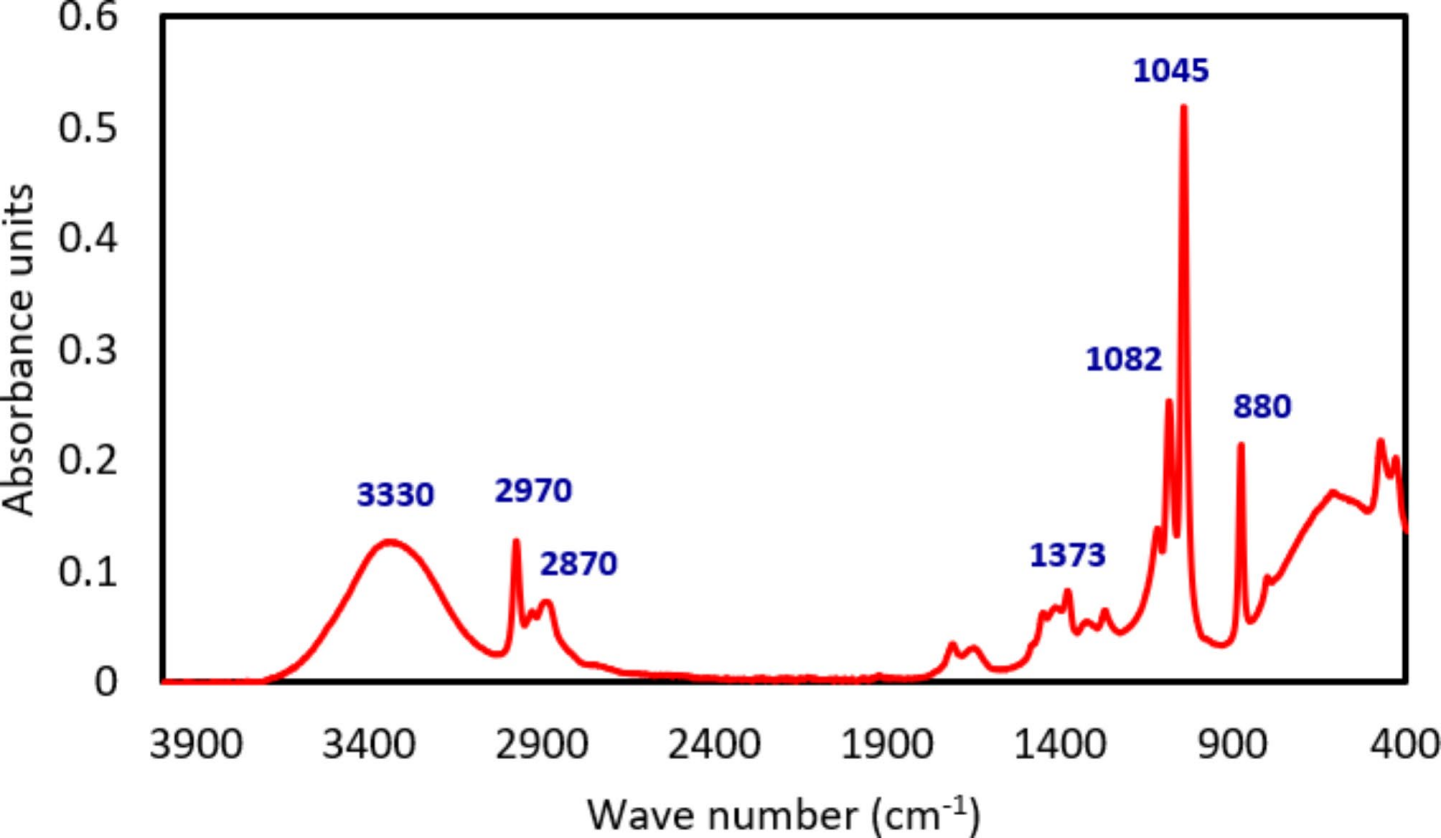
FTIR spectrum of the synthesised nano-coating liquid.

It is obvious from the elemental analysis that the nano-coating is a suspension of nano SiO_2_. To determine the nature of the carrying liquid, FTIR analysis was carried out and the results are presented in Fig. 5. The FTIR spectrum was identified by the software database as typical for pure ethanol with the characteristic O–H stretching peak at 3330 cm^−1^. The peaks at 1045, 1082, and 1373 cm^−1^ represent C–O stretching. The C–H stretching of the hydrocarbon chain appears at 2870 and 2970 cm^−1^.

#### Particle size and zeta potential

**Fig. 6:**
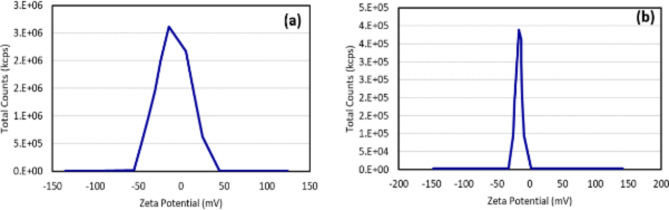
Zeta potential of (**a**) synthesised nano-coating and (**b**) dust particles.

The particle size analysis of the nano-coating showed that the average particle size is 55 nm which confirmed the nanometer nature of the coating, while the average particle size of the dust was 800 nm indicating the micrometer nature of the dust which facilitates its cleaning. The zetapotential analysis of the dust samples, Fig. 6b, revealed that dust is negatively charged with a net charge of – 17.5 mV. Consequently, increasing the net negative charge of the glass surface will minimize the dust adhesion. Figure 6a shows that the coated glass surface acquired a net negative charge of – 8.05 mV. The increase in the negativity of the glass surface is believed to be one of the anti-soiling mechanisms of the nano-coating.

#### Surface wettability

**Fig. 7:**
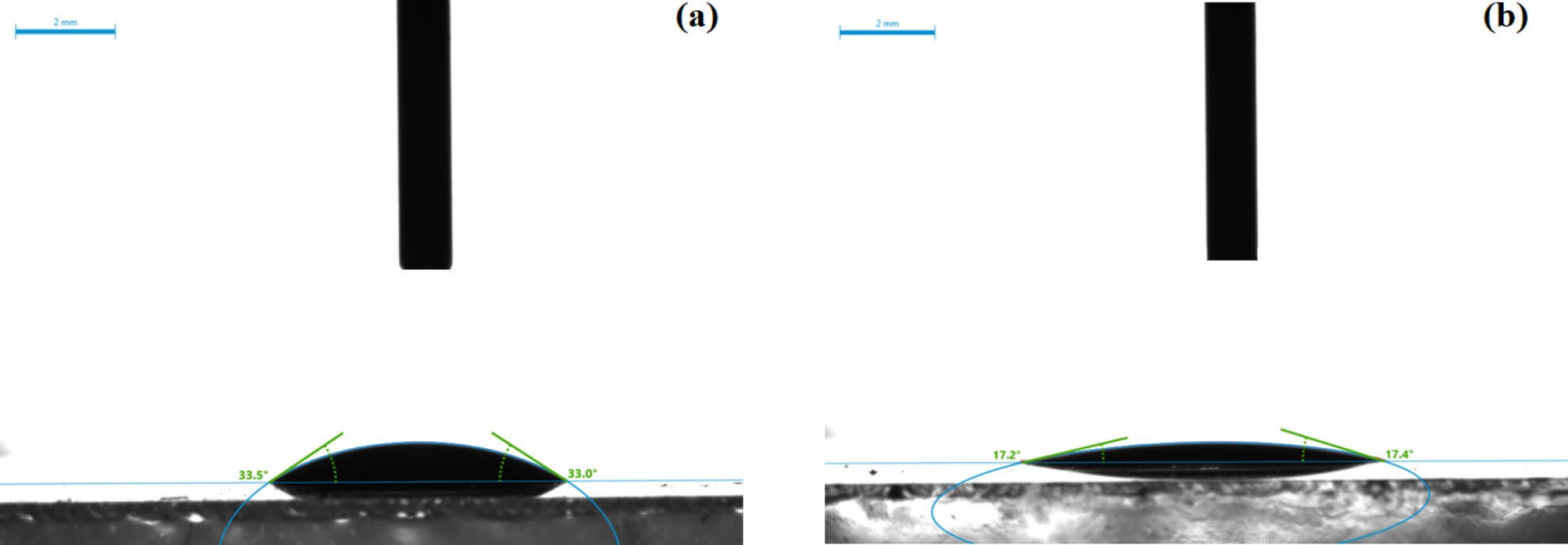
Water contact angle of (**a**) Bare glass substrate and (**b**) coated glass.

The surface wettability is a limiting factor in the anti-soiling properties of the glass surface. The approach commonly adopted in the literature is to render the surface either hydrophilic or hydrophobic. Therefore, the water contact angle of the glass surface was measured before and after the application of the nano-coating. As shown in Fig. 7, the bare glass substrate had a water contact angle of ~ 33° and when the nano-coating was applied the contact angle was halved. It has been reported that metal oxide coatings are generally utilized as transparent superhydrophilic coatings that enhance the self-cleaning ability of the surface by boosting the amount of hydroxyl groups that attract water molecules to the surface^38^. The lowered contact angle improves the diffusion of liquid water onto a surface which is considered an anti-soiling mechanism. If the surface of the PV module is properly inclined, the water droplets infiltrated under the deposited dust layer would carry the dust particles and slide away from the surface^36^. Considering a superhydrophobic surface, the self-cleaning mechanism depends upon the drops’ minuscule contact. Whereas a superhydrophilic surface is substantial for the flux of the liquid film and usually requires the presence of liquid flow to wash off the dust particles. Dust settlement on PV glass surfaces is mainly dependent on the local environment, including dust-type properties and no one solution fits all. Yet, in wet regions like the Egyptian climate, the proposed nano-coating is promising^39^. Several studies indicated that the adhesive forces of dust particles are akin to superhydrophilic and superhydrophobic surfaces. However, the anti-soiling performance of superhydrophilic coatings exhibited up to 2.5 times enhancement as compared to superhydrophobic coatings in outdoor field testing. Therefore, superhydrophilicity in such cases turned out to be more effective in reducing organic dirt and dust-moisture cementation-based soiling on the glass surface^39–41^.

### Performance of the PV panels and coating effectiveness

**Fig. 8:**
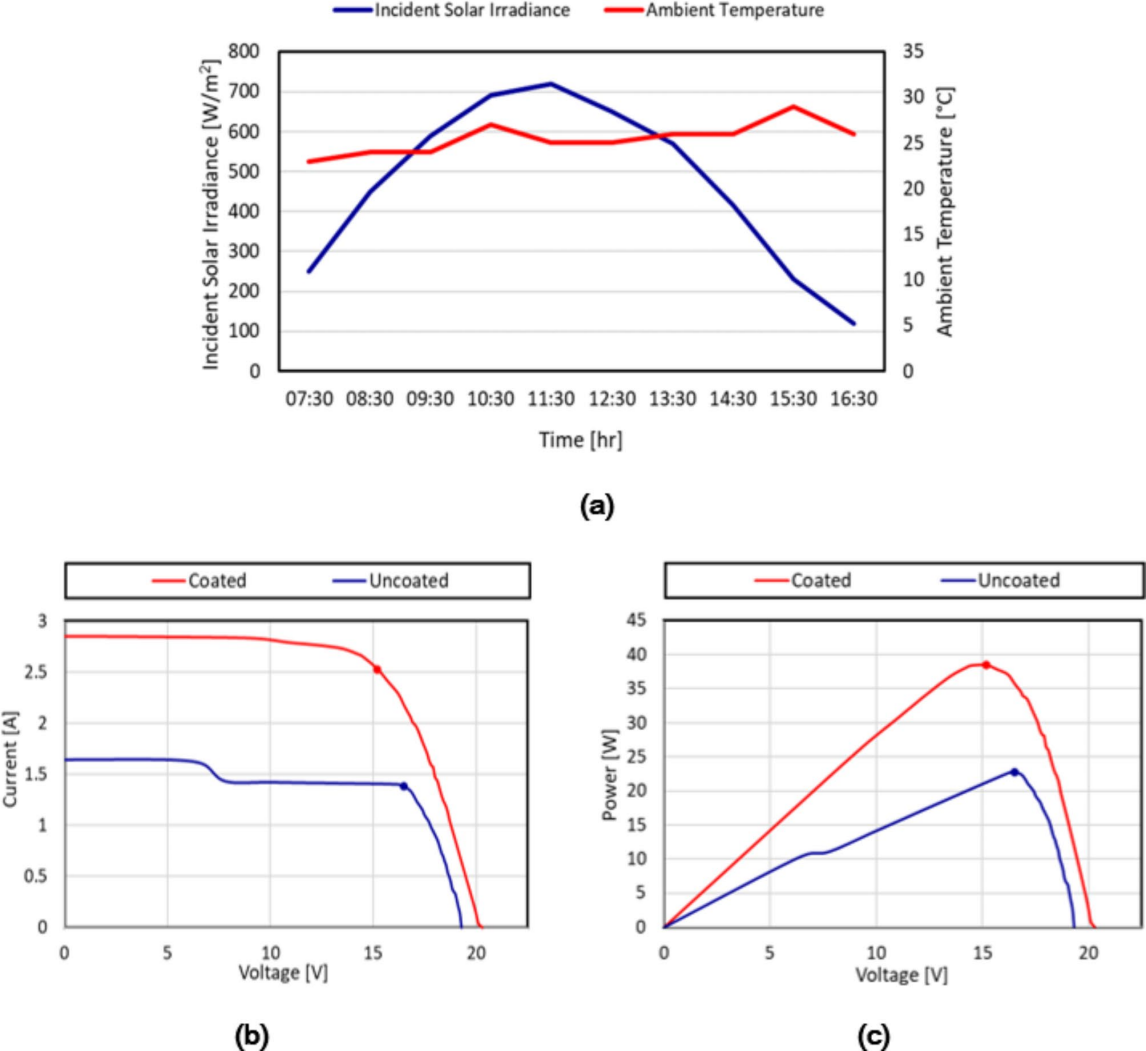
(**a**) Variation of the incident solar irradiance and ambient temperature throughout the day, (**b**) the I-V curve, and (**c**) the P-V curve of the coated and uncoated PV panels.

This section presents a comprehensive performance analysis of PV panels with and without the application of a synthesised thin film nano-coating. The analysis focuses on panels installed in Port Fouad, Port Said, Egypt [31^o^ 16 N, 32^o^ 18 E]. Conducted in June, the examination aimed to evaluate the coating’s impact on the panel’s overall performance. To ensure that the performance improvements observed are primarily due to the nano-coating, control variables such as the angle of incidence, metrological conditions, and duration of exposure were carefully managed throughout the experiment by placing both the PV panels with and without nano-coating at the same location in the vicinity of each other. Hence, the potential impact of the control variables on the results is minimized, thereby isolating the effect of the nano-coating on PV panel performance. Figure 8a provides a graphical representation of the measured solar irradiances and ambient temperature recorded throughout the day. The solar irradiance ranged from 120 W/m^2^ to a peak of 720 W/m^2^ at noon, while the ambient temperature recorded fluctuated between 23 °C and 29 °C. These data are crucial for understanding the environmental conditions under which the performance analysis was conducted. The $\:I{-}V$ and $\:P{-}V$ curves obtained for both the coated and uncoated panels provided valuable insights into the enhanced performance of the self-cleaning thin film nano-coating panel under Egyptian outdoor conditions. Figure 8b presents the $\:I{-}V$ curves for the coated and uncoated PV panels under identical solar irradiance conditions. It is evident from the curves that the coated panel consistently exhibited higher short circuit current ($\:{I}_{sc}$) values compared to the uncoated panel. The average $\:{I}_{sc}$ for the coated panel was 2.8 A, whereas, for the uncoated panel, it was 1.7 A. This notable increase (64.7%) in the $\:{I}_{sc}$ can be attributed to the effective dust removal capabilities of the self-cleaning nano-coating thin film, which prevented dust accumulation and minimized shading effects on the panel surface. The $\:P{-}V$ curves, as shown in Fig. 8c, further support the superior performance of the coated panel. The coated panel consistently demonstrated higher power outputs across the entire voltage range compared to the uncoated panel. The peak midday suggests optimal conditions for maximum energy production, which aligns with the observed performance metrics. The measured dust concentration on the uncoated panel amounted to 12 g/m^2^. The maximum power ($\:{P}_{max}$) achieved by the coated panel was 38 W, whereas, for the uncoated panel, it was 23 W. This improvement (65.2%) in $\:{P}_{max}$ can be attributed to the reduced resistive losses due to the enhanced surface cleanliness and reduced shading effects provided by the self-cleaning nano-coating thin film. The observed improvements in short circuit current and power production can be attributed to the self-cleaning nano-coating thin film properties, which effectively prevent the adhesion of dust particles on the panel surface. As a result, the coated panel experienced reduced dust accumulation and minimized surface contamination, leading to improved light absorption, and reduced resistive losses within the PV panel. In both the $\:I-V$ and $\:P-V$ curves, it is observed that there is a sudden decline in the values of the current and power of the uncoated panel indicating the detrimental impact of the dust shading effect on PV panel performance. Dust accumulation on the panel surface created shadows, reducing the effective surface area for light absorption. This resulted in a decrease in both power and current outputs. As illustrated in Fig. 8b and c, the electrical characteristics of the uncoated PV module displayed multiple peaks, resembling those seen in PV panels subjected to partial shading conditions. This occurrence arises from irregular dust deposition on the panel, affecting the lifespan of PV panels. Consequently, at the PV system level, non-uniform aging of PV panels distorts the electrical characteristics of the entire array, leading to significant power losses due to mismatch. This permanent mismatch accelerates the non-uniform aging, akin to a cascading effect, of PV panels which is a common problem in PV systems. Additionally, the distorted PV characteristics of the uncoated panels require sophisticated maximum power point tracking techniques to capture the maximum available power from the PV panel and avoid misleading power losses. This increases the complexity of the PV control scheme^42^.

**Fig. 9:**
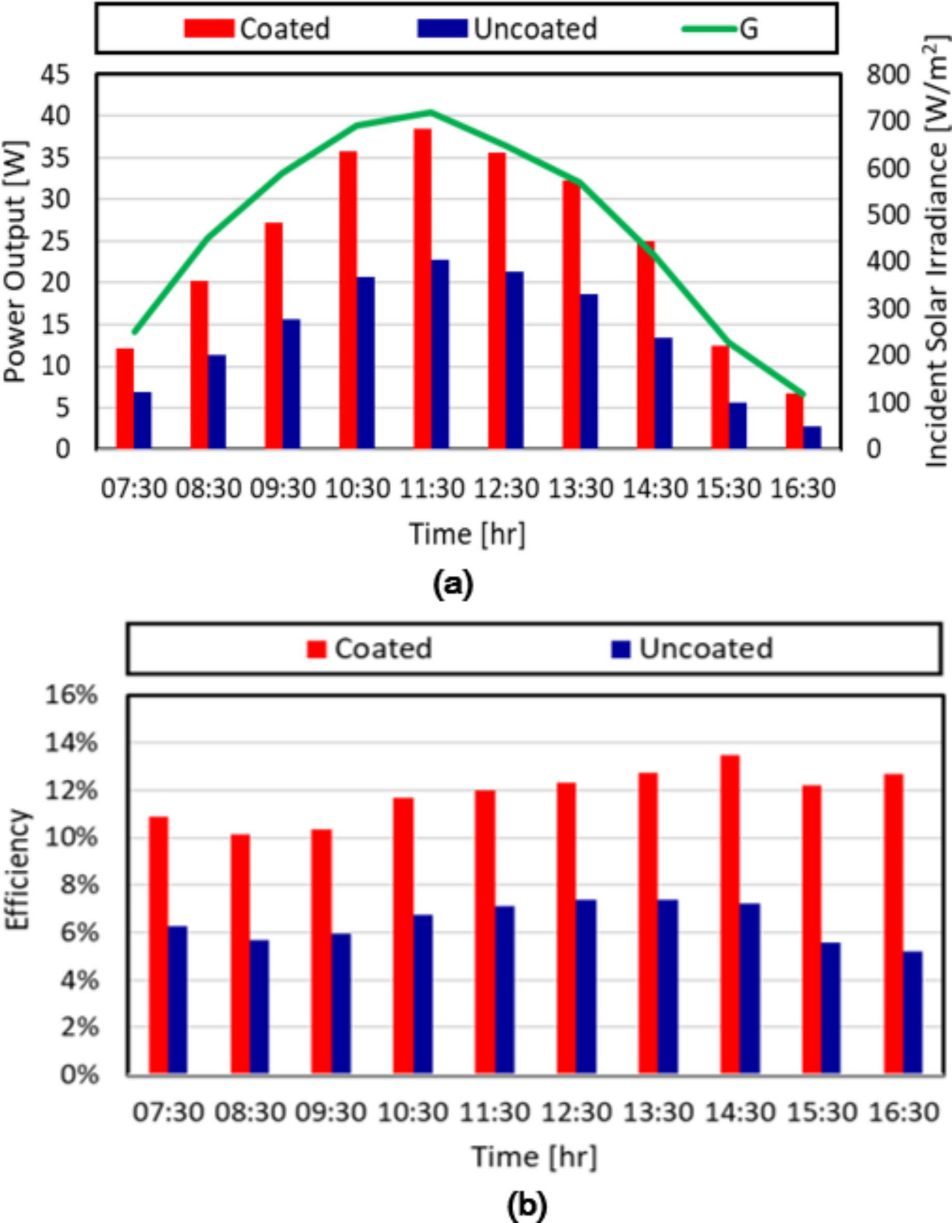
Hourly variation of (**a**) the maximum power output of both the coated and uncoated PV panels vs. the incident solar irradiance and (**b**) the efficiency of both the coated and uncoated PV panels.

The power production of the coated and uncoated PV panels throughout the day is illustrated in Fig. 9a. The power output of the coated panel ranged from 7 W to 38 W, with an average power generation of approximately 24.75 W. On the other hand, the uncoated panel produced between 3 W and 23 W, averaging around 14 W. These results indicate that the coated panel generated, on average, nearly twice as much power as the uncoated panel.The hourly data further highlights the superior performance of the coated panel. At each time interval, the power output of the coated panel exceeded that of the uncoated panel. For instance, at 10:30 AM, the coated panel produced 36 W compared to the uncoated panel’s 21 W. Similarly, at 12:30 PM, the coated panel generated 36 W, while the uncoated panel produced 22 W.The consistent trend of higher power generation from the coated panel can be attributed to the protective coating’s ability to enhance the panel’s efficiency and performance. The coating likely reduces losses due to reflection, improves light absorption, and minimizes the effects of dust and dirt accumulation on the panel surface. Moreover, silicon dioxide film function as passivation coatings, which improves the efficiency and performance of solar cells by minimizing electron-hole recombination. In addition, the passivation layer protects the solar cells from environmental factors consequently improving the longevity and stability of the cell^43^. Together, these elements contribute to an enhanced transformation of solar energy into electrical power. It is worth noting that the power output of both types of panels followed a gradual decline over the observation period. This decline can be attributed to various factors, such as the angle of incidence of sunlight and changes in solar radiation intensity. Figure 9b shows a comparison of the efficiency (ratio of the power output to the incident solar power) of both the coated and uncoated PV panels at various time intervals from 07:30 to 16:30. The coated panel consistently outperformed the uncoated panel throughout the day. During peak sunlight hours, the coated panel exhibited significantly higher efficiencies, ranging from 12 to 13.5% approaching the optimum efficiency provided by the manufacturer, compared to the uncoated panel’s stagnant efficiencies of 7–8%. By the end of the day, the coated panels still maintained an efficiency of 13% while the uncoated panels dropped to 5%. These results highlight the effectiveness of the coating in improving PV panel performance and energy conversion efficiency, particularly under optimal sunlight conditions.

## Conclusion

This research conducted an experimental investigation of the effectiveness of a self-cleaning nano-coating thin film in reducing dust buildup on photovoltaic (PV) panels in harsh climatic regions. The composition of each sample was analysed using Scanning Electron Microscopy (SEM), Energy-Dispersive X-ray spectroscopy (EDX), and Fourier Transform Infrared Spectroscopy (FTIR) to understand the dust and coating characteristics. Zeta potential testing and surface wettability analysis were also carried out. Outdoor experiments were conducted on panels installed in Egypt, revealing a significant improvement in panel performance with the coating. The coated panel exhibited a 64.7% increase in short circuit current and a 65.2% increase in maximum power compared to the uncoated panel. This translated to almost double the average power generation throughout the day for the coated panel. Moreover, the coated panel consistently achieved higher efficiencies, reaching peak values of 12–13.5% compared to the uncoated panel’s 7–8%. The nanocoating reduced dust adhesion, reducing shading effects and improving light absorption. These findings demonstrate the potential of nanocoating as an effective technique for dust mitigation and enhancing PV system efficiency, specifically in dusty environments. The ability of the coating to enhance efficiency by reducing reflection losses, improving light absorption, and minimizing the impact of dust accumulation were identified as key contributing factors. Despite these promising results, it is important to acknowledge the limitations of this study. The research was conducted at a single location in Egypt, and dust composition and environmental conditions can vary significantly in other regions. Future studies would assess the performance of the coating across diverse dusty environments. Additionally, the long-term durability and cost-effectiveness of the coating require further investigation. Incorporating wind speed as a variable in future research would provide a more comprehensive understanding of the nano-coating’s effectiveness under real-world conditions. These considerations are essential for optimizing and implementing this technology in practical applications. Overall, the results of this study strongly support the application of the nano-coatings thin film as a viable approach to improving the efficiency of photovoltaic panels in dusty environments.

## Data Availability

All datasets generated for this study are included in the manuscript.

## References

[1] Sulaiman, S. A., Singh, A. K., Mokhtar, M. M. M. & Bou-Rabee, M. A. Influence of dirt accumulation on performance of PV panels. *Energy Procedia* **50**, 50–56 (2014).

[2] Ahmed, A., Elsakka, M., Elminshawy, N., Mohamed, A. & Sundaram, S. Recent Advances in floating photovoltaic systems. *Chem. Record* (2023). 10.1002/tcr.202300229 37823687

[3] Chanchangi, Y. N., Ghosh, A., Sundaram, S. & Mallick, T. K. Dust and PV performance in Nigeria: A review. *Renew. Sustain. Energy Rev.* **121**, 109704 (2020).

[4] Enaganti, P. K. et al. Experimental investigations for dust build-up on low-iron glass exterior and its effects on the performance of solar PV systems. *Energy*. **239**, 122213 (2022).

[5] Ilse, K. *et al.* Techno-Economic Assessment of soiling losses and mitigation strategies for Solar Power Generation. *Joule* **3**, 2303–2321 (2019).

[6] Jamil, W. J., Rahman, A., Shaari, H. & Salam, Z. Performance degradation of photovoltaic power system: Review on mitigation methods. *Renew. Sustain. Energy Rev.* **67**, 876–891 (2017).

[7] Micheli, L., Fernandez, E. F. & Almonacid, F. Tracking soiling losses and cleaning profits trends, in *IEEE 48th Photovoltaic Specialists Conference (PVSC)* 0144-0146 (IEEE, 2021). (2021).

[8] Salamah, T. et al. Effect of dust and methods of cleaning on the performance of solar PV module for different climate regions: Comprehensive review. *Sci. Total Environ.* **827**, 154050 (2022). 35217056 10.1016/j.scitotenv.2022.154050

[9] He, B., Lu, H., Zheng, C. & Wang, Y. Characteristics and cleaning methods of dust deposition on solar photovoltaic modules-A review. *Energy*. **263**, 126083 (2023).

[10] Ekinci, F. et al. Experimental investigation on solar PV panel dust cleaning with solution method. *Sol. Energy*. **237**, 1–10 (2022).

[11] Aljaghoub, H., Abumadi, F., AlMallahi, M. N., Obaideen, K. & Alami, A. H. Solar PV cleaning techniques contribute to Sustainable Development Goals (SDGs) using multi-criteria decision-making (MCDM): Assessment and review. *Int. J. Thermofluids*. **16**, 100233 (2022).

[12] Mozumder, M. S., Mourad, A. H. I., Pervez, H. & Surkatti, R. Recent developments in multifunctional coatings for solar panel applications: a review. *Sol. Energy Mater. Sol. Cells*. **189**, 75–102 (2019).

[13] Midtdal, K. & Jelle, B. P. Self-cleaning glazing products: A state-of-the-art review and future research pathways. *Sol. Energy Mater. Sol. Cells* **109**, 126–141 (2013).

[14] Tayel, S. A., El-Maaty, A., Mostafa, A. E. & Elsaadawi, Y. F. E. M. Enhance the performance of photovoltaic solar panels by a self-cleaning and hydrophobic nanocoating. *Sci. Rep.* **12**, 1–13 (2022). (2022). 10.1038/s41598-022-25667-4 PMC9732349 36481954

[15] SelfCleaning Solar Panels Maximize Energy Efficiency - ASME. https://www.asme.org/topics-resources/content/self-cleaning-solar-panels-maximize-efficiency

[16] Lei, H. *et al.* Superhydrophobic coatings based on colloid silica and fluorocopolymer. *Polymer (Guildf)* **86**, 22–31 (2016).

[17] Das, A. et al. Superhydrophobic and conductive carbon nanofiber/PTFE composite coatings for EMI shielding. *J. Colloid Interface Sci.* **353**, 311–315 (2011). 20889160 10.1016/j.jcis.2010.09.017

[18] Zuo, Z., Gao, J., Liao, R., Zhao, X. & Yuan, Y. A novel and facile way to fabricate transparent superhydrophobic film on glass with self-cleaning and stability. *Mater. Lett.* **239**, 48–51 (2019).

[19] Washeem, M. *et al.* Super hydrophilic surface coating for PV modules. *Green. Energy Technol.* 185–209 (2022).

[20] Elnozahy, A., Abd-Elbary, H. & Abo-Elyousr, F. K. Efficient energy harvesting from PV panel with reinforced hydrophilic nano-materials for eco-buildings. *Energy Built Environ.* (2022).

[21] Wang, Y. *et al.* Fabrication of nanostructured CuO films by electrodeposition and their photocatalytic properties. *Appl. Surf. Sci.* **317**, 414–421 (2014).

[22] Jiang, T. et al. Controllable fabrication of CuO nanostructure by hydrothermal method and its properties. *Appl. Surf. Sci.* **311**, 602–608 (2014).

[23] Perkas, N., Amirian, G., Girshevitz, O. & Gedanken, A. Hydrophobic coating of GaAs surfaces with nanostructured ZnO (2016).

[24] Qing, Y., Yang, C., Hu, C., Zheng, Y. & Liu, C. A facile method to prepare superhydrophobic fluorinated polysiloxane/ZnO nanocomposite coatings with corrosion resistance. *Appl. Surf. Sci.* **326**, 48–54 (2015).

[25] Egatz-Gómez, A. et al. Silicon nanowire and polyethylene superhydrophobic surfaces for discrete magnetic microfluidics. *Appl. Surf. Sci.* **254**, 330–334 (2007).

[26] Nasser, M. & Hassan, H. Egyptian green hydrogen Atlas based on available wind/solar energies: power, hydrogen production, cost, and CO2 mitigation maps. *Int. J. Hydrog. Energy* **50**, 487–501 (2024).

[27] Bassam, A. M. et al. Conceptual design of a novel partially floating photovoltaic integrated with smart energy storage and management system for Egyptian North Lakes. *Ocean Eng.* **279**, (2023).

[28] Moharram, N. A., Tarek, A., Gaber, M. & Bayoumi, S. Brief review on Egypt’s renewable energy current status and future vision. *Energy Rep.* **8**, 165–172 (2022).

[29] Shenouda, R., Abd-Elhady, M. S. & Kandil, H. A. A review of dust accumulation on PV panels in the MENA and the Far East regions. *J. Eng. Appl. Sci.* **69**, 1–29 (2022).

[30] Menoufi, K., Mohamed, H. F. M., Farghali, A. A. & Khedr, M. H. Dust accumulation on photovoltaic panels: A case study at the East Bank of the Nile (Beni-Suef, Egypt). *Energy Procedia* **128**, 24–31 (2017).

[31] Elminir, H. K. et al. Effect of dust on the transparent cover of solar collectors. *Energy Convers. Manag*. **47**, 3192–3203 (2006).

[32] Al-Badra, M. Z., Abd-Elhady, M. S. & Kandil, H. A. A novel technique for cleaning PV panels using antistatic coating with a mechanical vibrator. *Energy Rep.* **6**, 1633–1637 (2020).

[33] Alamri, H. R., Rezk, H., Abd-Elbary, H., Ziedan, H. A. & Elnozahy, A. Experimental investigation to improve the energy efficiency of solar PV panels using hydrophobic SiO2 nanomaterial. *Coat* **10**(10), 503 (2020).

[34] Ma, H. P. et al. Annealing effect on SiNx/SiO2 superlattice with ultrathin sublayer fabricated using plasma-enhanced atomic layer deposition. *Ceram. Int.* **48**, 22123–22130 (2022).

[35] Ismail, A. A. et al. Self-cleaning coatings for minimizing the impact of dust precipitation on the power production of solar cells utilizing mesoporous TiO2/SiO2 and ZnO/SiO2 films. *Ceram. Int.* **49**, 22788–22796 (2023).

[36] Adak, D., Bhattacharyya, R. & Barshilia, H. C. A state-of-the-art review on the multifunctional self-cleaning nanostructured coatings for PV panels, CSP mirrors and related solar devices. *Renew. Sustain. Energy Rev.* **159**, 112145 (2022).

[37] Attar, A. et al. Fabrication, characterization, TD-DFT, optical and electrical properties of poly (aniline-co-para nitroaniline)/ZrO2 composite for solar cell applications. *J. Ind. Eng. Chem.* **109**, 230–244 (2022).

[38] Gao, Y., Liu, J. & Dong, B. Improvement of the dust removal performance of a high-transmittance SnO2–SiO2 film by Sb doping. *Ceram. Int.* **47**, 8677–8684 (2021).

[39] Jesus, M. A. M. L. *et al.* Anti-soiling coatings for solar cell cover glass: Climate and surface properties influence. *Sol. Energy Mater. Sol. Cells* **185**, 517–523 (2018).

[40] Jang, G. G. et al. Transparent superhydrophilic and superhydrophobic nanoparticle textured coatings: comparative study of anti-soiling performance. *Nanoscale Adv.* **1**, 1249–1260 (2019). 36133208 10.1039/c8na00349a PMC9473203

[41] Syafiq, A., Pandey, A. K., Adzman, N. N. & Rahim, N. A. Advances in approaches and methods for self-cleaning of solar photovoltaic panels. *Sol. Energy* **162**, 597–619 (2018).

[42] Refaat, A., Osman, M. H. & Korovkin, N. V. Current collector optimizer topology to extract maximum power from non-uniform aged PV array. *Energy*. **195**, 116995 (2020).

[43] Arul Prishya, A. S. & Chopra, L. Manikanika. Comprehensive review on uses of silicon dioxide in solar cell. *Mater. Today Proc.* **72**, 1471–1478 (2023).

